# Nurses’ Establishment of Health Promoting Relationships: A Descriptive Synthesis of Anorexia Nervosa Research

**DOI:** 10.1007/s10826-016-0534-2

**Published:** 2016-09-13

**Authors:** Martin Salzmann-Erikson, Jeanette Dahlén

**Affiliations:** 1 Faculty of Health and Occupational Studies, Department of Health and Caring Sciences, University of Gävle, Gävle, SE-80176 Sverige Sweden; 2 Child and Adolescent Psychiatry Outpatient Clinic, Stockholm County Council, Stockholm, Sweden, Ersta Sköndal University College, Institution for Caring Science, Stockholm, Sweden

**Keywords:** Anorexia nervosa, Literature review, Psychiatric nursing, Relationship

## Abstract

Qualitative values that address personal and interpersonal dimensions are often overlooked in research that examines mental well-being among young patients with anorexia nervosa. The aim of this review was to identify and describe factors that promote and impede the relationships between nurses and the children, adolescents and young adults who are diagnosed with anorexia nervosa and also to explore and describe how those relationships benefit the patients’ processes toward increased health and well-being. A descriptive literature synthesis was conducted following the four steps as described by Evans. The three databases CINAHL, PsycINFO and PubMed were used to search for qualitative articles. Fourteen articles met the criteria for inclusion and were analysed. Key findings were identified, and categories and themes were formulated and compared across the studies. Four themes are presented in the results: (1) The essentials in a relationship; (2) The person at the centre; (3) The nurses’ attitudes; and (4) Knowledge. In addition to the contribution to the knowledge of how anorexia is manifested, our findings demonstrate the necessity for nurses to be person-centred in their relationships with patients and to have attitudes characterised by presence, genuine commitment and motivation. Nurses are more likely to convey a sense of trust and safety when they communicate with openness and honesty. Our review suggests that the motivation for patients to adhere to treatment is likely to increase when nurses approach patients with these characteristics and attitudes. We argue that the findings are relevant for nurses in their everyday practices.

## Introduction

Anorexia nervosa (AN) primarily affects adolescent girls and young women and is characterised by excessive dieting, leading to severe weight loss with a pathological fear of becoming fat or unhealthy behaviours that hinder weight gain despite clear indications of remaining underweight. Another aspect of the disease is a distorted body image, affecting ideas about weight and body form, along with a self-image that is overly influenced by these factors (American Psychiatric Association 2013).

A longitudinal study from the U.S. showed that the mortality among individuals with AN is significantly higher than that of the general population (Franko et al. 2013). Two meta-analyses have also shown that AN has the highest mortality among all psychiatric diseases (Arcelus et al. 2011; Smink et al. 2012). Causes for death are most often physiological factors, such as undernourishment and cardiovascular collapse (Wilkes and Anderson 2000), but suicide also occurs often among youth with AN (Papadopoulos et al. 2009). AN usually debuts during an important and identity-forming time in life when the individuals are between 15–20 years old. The onset has serious consequences for the youth themselves, and it also has extensive consequences for the whole family and their living conditions (Russell 2006). Reasons for onset are not completely mapped out, but predispositions, such as family, socio-cultural, genetic, biological and individual factors have been identified (Hällström 2010; Nilsson et al. 2007). A Danish cohort study that followed over 2300 children and youth for three generations showed that there is a heightened risk for the development of AN within families that have incidences of affective disorders, including anxiety, obsessive-compulsive disorder, personality disorders and substance abuse as well as those with siblings suffering from AN (Steinhausen et al. 2015).

Eating disorders are often reported to be difficult to treat, and caring for patients diagnosed with AN is often complex due to low motivation for treatment among the patients (Geller et al. 2001; Marzola et al. 2012; Vitousek et al. 1998). Beyond the fear of gaining weight or losing control (Palmer 2000), a negative self-image is also said to be characteristic for this patient group, all of which contribute to difficulties in treatment (Björck 2006). Higher levels of guilt and hate tied to the person as well as lower levels of self-affirmation and self-love are shown in people with AN in comparison with people with other eating disorders. AN is reported to be the eating disorder that is tied most closely with self-control (Björck 2006).

One reason that youths with AN are ambivalent and choose to fight treatment of their illness can be the positive effects they experience using the disease and its restrictions as tools for control and handling of emotions-something treatment would prevent (Treasure et al. 1999; Williams and Reid 2010). Giving oneself over to treatment is about letting go of control, and therefore complexity in care arises (Palmer 2002; Paulson Karlsson 2012). In treatment of patients with high degrees of self-hate, there is a risk that those treating the patient respond to the patient in a negative way. Patients with a high degree of self-hate can provoke hostile reactions from therapists, and low levels of rejection, accusations and belittlement can negatively influence the treatment (Björck 2006; Kiesler 1996).

The establishment of emotional alliances and trusting relationships is essential in psychiatric care (Borg and Kristiansen 2004; Denhov and Topor 2012; Peplau 1991). The relationship between nurses and patients is paramount in psychiatric nursing in order to accomplish a change that will help and direct patients’ processes toward health and recovery (Barker and Buchanan-Barker 2005; Dahlberg and Segesten 2010; Shanley and Jubb-Shanley 2007). In a prospective cohort study of adolescents with AN in France, Bourion-Bedes et al. (2013) demonstrated the correlation between AN patients’ perceptions of early therapeutic alliances and shorter times in achieving target weights. Other studies report on the way motivation towards changing eating habits varies among patients with AN (Nordbo et al. 2011; Vansteenkiste et al. 2005).

In order to support progress in the treatment of AN, it is essential to explore and address patients’ expressed motivations at an early stage and throughout the process to help patients maintain a positive attitude toward change (Paulson Karlsson 2012). According to Pereira et al. (2006), difficulties in creating an alliance with adolescents with AN are often due to a strong identification with the disease, low cognitive functioning related to starvation and being in treatment because the parents have demanded it, not because they have decided to seek treatment on their own. Earlier studies also show that parents often feel a stronger alliance with caregivers than the adolescents in treatment do. This had an impact on treatment progress, as patients did not feel secure and felt less positive toward change (Forsberg et al. 2013; Halvorsen and Heyerdahl 2007; Isserlin and Couturier 2012). However, parents’ alliances with caregivers are important for their roles as parents and their further support of their children after discharge (Honey et al. 2007; Isserlin and Couturier 2012). According to McCormack and McCance (2006), the foundation in person-centred care is comprised of different constructs, and one important construct is the attributes of the nurse, including being professionally competent and interpersonally skilled. The literature generally emphasises what a health-promoting relationship is and how it can influence patients on their path to good health (Dahlberg and Segesten 2010; McCormack and McCance 2006; Safran and Muran 2000). However, there is a lack of clear descriptions of specific factors that contribute to creating this important relationship between nurses and patients with AN.

To promote the development of the clinical work that aims to reduce suffering during care, reduce the length of care and increase the results of treatment, there is a strong incentive to gather knowledge that identifies the factors that promote a care relationship that supports the patient’s health process. We believe that nurses hold a position in which they are able to impact the quality of care because of their abilities to manage the complexity in their relationships with patients. Hence, we conducted a descriptive literature synthesis in which our aim was to identify and describe factors that promote and impede the relationships between nurses and children, adolescents, and young adult patients with AN. Our aim was to also explore and describe how those relationships benefit the patients’ processes toward increased health and well-being.

## Method

In this review we analysed and synthesised original research based on subjective experiences concerning relationships between nurses and patients. This descriptive literature synthesis is grounded in our idea that reality is socially constructed, as opposed to the positivist paradigm. Hence, the focus in this review accounts for ‘studying people in natural settings while engaging in life experiences’, also termed naturalistic enquiry (Lincoln and Guba 1985). In order to create a systematic structure of the research process we chose to adopt the process for synthesising qualitative data outlined by Evans (2002). In summary, Evans identifies the following steps in this process: (1) Gather the sample, (2) Identify the key findings, (3) Categorise themes across studies, and (4) Describe the phenomena.

Step 1: According to Evans (2002) we determined the unit of analysis and which studies to include and exclude. The inclusion criteria were the following: original peer-reviewed articles on qualitative studies, written in English, published between 2004–2014, relevant to the purpose of the review, addressing children, adolescents and young adults (no specific age limits), scored as middle or high quality (evaluating the studies), and with a clear focus on patients diagnosed with AN and nurses’ experiences of caring for patients diagnosed with AN. Next, we determine which databases should be used. In collaboration and discussion with the librarian at the university, three databases were chosen due to their appropriateness in relation to the aim of the review: CINAHL, PsycINFO and PubMed. Search terms varied slightly across the databases. In summary, the following terms were used: CINAHL (search terms as “Subject Headings”: anorexia nervosa, qualitative studies, nursing care, interpersonal relationships, professional-patient relationships, Manual search: anorexia, qualitative, nurse*, relationship*, therapeutic alliance, effective nursing*). Six hundred eighty articles were found and all titles were read, out of those, 41 abstracts were read, 22 of the articles were reviewed and finally 12 chosen for inclusion. PsycINFO (search terms as Thesaurus: anorexia nervosa, qualitative research, therapeutic process*, interpersonal relationship*, Manual search: anorexia nervosa, nursing, therapeutic relationship*, alliance, effective nursing, professional relationships*). Three hundred fifty articles were found and all titles were read, out of those, 11 abstracts read, 6 articles reviewed and one chosen for inclusion. PubMed (search terms with MESH: anorexia nervosa, nurse-patient relationship*, professional-patient relationship*, qualitative research). Three hundred fifty four articles were found and all titles were read, out of those, four abstracts were read, one articles were reviewed but no article was chosen for inclusion. In addition, a manual search was conducted based on the reference list in the identified articles; the manual search resulted in one article chosen to be included in the review. See (Supplementary Table 1) for more details about search terms, combinations and number of hits. Since this is a review, permission to include the articles were not required.

Next, we selected the sample based on the search. First, the titles of the articles were read and determined whether the study suited our review aim. Of those articles that were considered suitable, the whole abstracts were read. A purposeful sample of 30 articles were selected based on how well the results in the included studies answered the research aim. Four articles were excluded, as they were not relevant even though the abstract at first seemed relevant. The remaining 26 articles were quality-valued based on a review template (Supplementary Table 2). The two review templates published in Forsberg and Wengström (2013) and in Willman et al. (2006) were merged both into one. Hence, it was possible to cover all important aspects when reviewing a research article. In addition, a point system was added to the modified template in order to quantify the quality. The template consisted of 25 questions regarding the quality of the article, covering aim, methodology, results, credibility, clinical relevance and ethical considerations. Each question in the template could be answered with a yes, giving one point and no, giving zero points. The points were summarised for each article, and 0–17 points was considered to indicate low quality, 18–20, middle-range quality and 21–25 points indicated high-quality. Four articles were excluded due to low quality. The review was performed by the second author. After internal discussion between the authors, an additional eight articles were excluded due to low relevancy, as the results in the articles did not provide accurate data to answers to the research aim. The remaining 14 articles, involving a total participant number of 134 patients and 33 nurses, were included for analysis (See Table 1).

**Table 1:** Analyses of selected studies

	Author, year, country	Title	Purpose of the study	Design	Participants	Method of analysis	Main results and quality
A	Ross & Green (2011). England.	Inside the experience of anorexia nervosa: A narrative thematic analysis	To research if in-patient care is experienced as therapeutic	Qualitative. Semi-structured interviews.	2 patients (over 18 years old) that had been sick with AN for over five years, with in-patient experience. Now in specialised day patient clinic.	Thematic analysis	Stresses the importance of nurturing relationships in the treatment of chronically ill patients with AN.
							High. 22/25
B	Van Ommen et al. (2009). The Netherlands.	Effective nursing care of adolescents diagnosed with anorexia nervosa: the patients’ perspective	To describe effective nursing practices in in-patient care for patients with AN, from a patient’s perspective.	Qualitative. Semi-structured interviews.	13 patients (13–17 years old) that were treated in a specialised clinic with out-patient and in-patient care.	Grounded theory	Nurses contributed significantly to the recovery from AN through normalisation, structure and responsibility.
							High. 22/25.
C	Zugai et al. (2012). Australia.	Effective nursing care of adolescents with anorexia nervosa: a consumer perspective	To describe how nurses enable weight gain and a positive experience for adolescents in treatment for AN, from a patient’s perspective.	Qualitative. Semi-structured interviews.	8 patients (12–18 years old) with in-patient experience, but now in specialised day patient clinic.	Thematic analysis	Nurses’ characteristics were seen as having a strong influence on patients’ experiences, primarily regarding weight gain, maintaining a therapeutic environment and the relationship between nurses and patients.
							High.21/25
D	Gulliksen et al. (2012). Norway.	Preferred therapist characteristics in treatment of anorexia nervosa: the patient’s perspective	To describe which characteristics that patients with AN prefer in therapists.	Qualitative. Semi-structured interviews.	38 patients (18–51 years) that were treated as day patients, in-patients and out-patients at specialised clinics as well as general hospitals.	Thematic analysis	The care of patients with AN requires a caregiver with the ability to use a complex spectrum of skills. Preferred characteristics and skills included acceptance, vitality, expertise and the understanding to challenge the patient.
							High. 21/25.
E	Ramjan (2004). Australia.	Nurses and the ‘therapeutic relationship’: caring for adolescents with anorexia nervosa	To research the difficulties that prevent relationships between nurses and patients with AN.	Qualitative. Semi-structured interviews.	10 nurses with at least two years of experience treating patients in in-patient care at a general children’s hospital.	Thematic analysis	The main themes in the care of patients with AN were 1) the aim to understand the disease, 2) the pursuit of control and a balance of power with the patient, 3) the desire to build a caring relationship and the difficulty of creating alliances.
							High.22/25.
F	Offord et al. (2006). England.	Adolescent inpatient treatment for anorexia nervosa: a qualitative study exploring young adults’ retrospective views of treatment and discharge	To explore young adults’ opinions about previous in-patient care, how these experiences in care impact the need for control and low self-esteem as well as discharge.	Qualitative. Semi-structured interviews.	7 patients (16–23 years old) with experience with in-patient care.	Interpretive phenomenological analysis	Expressions of the adolescents’ normal development were not noted by the caregivers, which made things more difficult for the patients, and authoritative relationships were thought to contribute to feelings of isolation and inferiority.Medium high. 19/25.
G	Jenkins & Ogden (2011). England.	Becoming ‘whole’ again: a qualitative study of women’s views of recovering from anorexia nervosa	To explore how women experienced their recovery from AN.	Qualitative. Semi-structured in-depth interviews.	15 patients, (over 18 years old) in the recovery stage with experience with both in-patient and day patient care.	Interpretive phenomenological analysis	The relationships with professionals had an influence on the recovery process, specifically regarding irrational and rational thinking and behaviours
							Medium high. 19/25.
H	Micevski & McCann (2005). Australia.	Developing interpersonal relationships with adolescents with anorexia nervosa	To describe how nurses develop therapeutic relationships with adolescents with AN.	Qualitative. unstructured in-depth interviews.	10 nurses that treat patients with AN as in-patients in a children’s clinic.	Grounded theory	An individual focus and a sense of equality and respect are essential in care and for creating therapeutic relationships with patients with AN.
							Medium high. 19/25.
I	Bakker et al. (2011). The Netherlands.	Recovery of normal body weight in adolescents with anorexia nervosa: the nurse’s perspective on effective interventions	To research the most effective aspects in nursing with adolescents with AN regarding weight gain. From a nurse’s perspective.	Qualitative. Semi-structured in-depth interviews.	8 nurses & 1 social worker that treat patients between 12–18 years with AN in a specialised clinic.	Thematic analysis	The nurses saw themselves as in a key position to support patients in weight gain, and a good therapeutic relationship was the most essential aspect for this.
							Medium-high. 18/25.
J	Pemperton & Fox (2011). England.	The experience and management of emotions in an inpatient setting for people with anorexia nervosa: a qualitative study	To describe meaningful factors for caring for and aiding with managing emotions for people with AN in in-patient care.	Qualitative. Semi-structured interviews.	8 patients (under 25 years old) from two specialised units with in-patient care.	Interpretive phenomenological analysis	Some strategies among health professionals could aid in maintaining the anorexic behaviours in patients, while some anorexic behaviours in patients had negative impacts on the staff and therefore the care.
							Medium high. 20/25.
K	Sly et al. (2014). England.	Rules of engagement: qualitative experiences of therapeutic alliance when receiving in-patient treatment for anorexia nervosa	To explore patients’ experiences with developing therapeutic alliances during hospital stays.	Qualitative. Semi-structured in-depth interviews.	8 patients (18–34 years old), that were treated in day patient care with earlier experiences with in-patient care.	Interpretive phenomenological analysis	Alliances are a main component in the treatment of eating disorders, and they are influenced by trust, safety and a sense of equality.
							Medium high. 19/25.
L	Wright & Hacking (2012). England.	An angel on my shoulder: a study of relationships between women with anorexia and healthcare professionals	To describe the experience of the therapeutic relationship between women with AN and their caregivers and to examine the contexts that enable this.	Qualitative. Semi-structured in-depth interviews.	6 patients (21–44 years old), as well as their 7 caregivers (1 dietician, 1 therapist, 5 nurses). Patients were treated in day patient care and had been in different treatments for an average of 11 years.	Thematic analysis	Six topics that influence the caring relationship were presented: 1) sincerity, 2) safety, 3) the process of the disease, 4) recovery measured in kilograms, 5)hope and optimism, 6) the caregiver’s characteristics in handling patients.
							Medium high. 18/25.
M	Colton & Pistrang (2004). England.	Adolescents’ experiences of inpatient treatment for anorexia nervosa.	To describe the experience of in-patient care for adolescents with AN.	Qualitative. Semi-structured interviews.	19 patients (12–17 years old), treated at a specialised clinic. Many of the patients had experience with previous stays in in-patient care at specialised clinics and in general hospitals.	Interpretive phenomenological analysis	Patients experienced conflicts and dilemmas regarding treatment, insight into the disease and the desire to get well. Key aspects of this were described.
							High. 21/25.
N	Tierney (2008). England.	The individual within a condition: a qualitative study of young people’s reflections on being treated for anorexia nervosa.	To explore young people’s experiences of being treated for AN.	Qualitative. Semi-structured interviews.	10 patients that were treated for AN as teens (between 11–18 years old). Participants were in different stages of the disease and treatement process but had been in treatment within the last three years.	Thematic analysis	Aspects that patients considered important in care were discussed: the balance between the physical and the psychological, certain characteristics in professionals, and the experience of improvement in the process toward better health.
							High. 22/25.

Step 2: The analysis procedure described by Evans (2002) includes reading and collecting the findings. In order to conduct our inductive analysis, the included articles were read thoroughly, and notes were taken. Both the results in the original studies and conclusions were considered as data. Texts that were relevant to our purpose were coded with different colours, given a code number and pasted into a separate spreadsheet with five columns (Supplementary Table 3). The first column shows the author names, and the key findings are in the second column.

Step 3: In accordance with Evans’ (2002) descriptive synthesis process we reviewed the key findings and compared them with those in the other studies. We condensed the key findings to get a clear picture of the text, and these were written down in the third column, presented in (Supplementary Table 3). The text in the third column was also copied onto post-it notes. The post-it notes were placed on a wall and sorted according to their similarities and differences. This led to the creation of four main themes, which are noted in column four, after each key finding. The next step according to Evans (2002) is to search for nuances within the themes. The key findings within each theme were compared with each other. Based on the differences within the themes, we constructed different sub-themes. These were noted after each key finding in column five in the document. In total, we identified ten sub-themes which gave a nuanced and comprehensive understanding of the phenomena. Next, we compared and discussed the key-findings and the themes in order to view the parts in relation to the larger context in which they belonged. The content of the themes was reflected on using further comparisons with the articles’ outcomes and through a review of previously written notes and key findings. When two sub-themes tended to flow into each other, these were merged into one sub-theme.

Step 4: The fourth and final step in Evans’ (2002) synthesis process is to describe the phenomena. With reference to the qualitative studies, each of the four themes and corresponding sub-themes are explained below in the “Results” section.

## Results

The results are presented under the four themes: (1) The essentials in a relationship, including the sub-themes (a) Feelings of solidarity, participation and equality, (b) Openness, integrity and honesty and (c) Trust and safety; (2) The person at the centre, including the sub-themes (a) Seeing the person behind the diagnosis and (b) Balance between the physical and the psychological, (3) The nurses’ attitudes, including the sub-themes (a) Motivation and hope, (b) Maintaining structure, responsibility and normality and (c) Presence and availability; and (4) Knowledge, including the sub-themes (a) Understanding, experience and knowledge and (b) Emotional management and identification.

### The Essentials in a Relationship

This theme relates to factors that are the essence for establishing a relationship, as they promote a collaboration between the nurse and the patient and the patient’s health process. This is the overarching theme for the following three sub-themes: feelings of solidarity, participation and equality; openness, integrity and honesty; and trust and safety.

#### Feelings of Solidarity, Participation and Equality

Equality in the relationship and respect for each other’s positions as nurse and patient along with involvement in care were described as significant for the relationship (Colton and Pistrang 2004; Gulliksen et al. 2012; Offord et al. 2006; van Ommen et al. 2009; Sly et al. 2014). Equality in the relationship meant that patients felt that they could express their feelings as well as that the nurses were able to freely express what was expected from the patient. Several studies have further described the importance of nurses actively allowing the patients to be more involved to create an alliance (Colton and Pistrang 2004; Jenkins and Ogden 2011; van Ommen et al. 2009). Colton and Pistrang (2004) reported care without involvement, care that was done to or for the patients and not with them, as a negative experience. Furthermore, lack of involvement could lead to resistance and hamper the recovery process and the relationship and could even worsen the illness (Sly et al. 2014). The nurse’s ability to establish a personal connection in the relationship was described as important in the recovery process and essential for the establishment of a trusting relationship (Ross and Green 2011; Wright and Hacking 2012; Zugai et al. 2012). When nurses provided information about their own personal lives, they conveyed a sense of equality between themselves and the patient, which was considered important in the development of the relationship (Micevski and McCann 2005). However, the nurses felt that this kind of relationship could negatively affect patients’ willingness to open up if the nurses were too much in focus. Hence, a professional balance regarding the extent to which the nurses should be able to share information about themselves was emphasised.

#### Openness, Integrity and Honesty

The aspects of openness, integrity and honesty were identified as vital in establishing a relationship (Micevski and McCann 2005; van Ommen et al. 2009; Sly et al. 2014; Wright and Hacking 2012; Zugai et al. 2012). A genuine commitment of the nurses, the feeling that they were not just ‘doing a job’, contributed to a sense of safety among the patients (Gulliksen et al. 2012; Pemperton and Fox 2011; Wright and Hacking 2012; Zugai et al. 2012). In Pemperton and Fox’s (2011) study, the author writes that even though nurses showed empathy and a desire to validate the patient’s feelings, it was only when the nurses really showed a genuine commitment that patients felt meaning in care. Such commitment made it possible for the patient to see beyond the role of the nurse only as a professional, which promoted an individual and unique relationship (Pemperton and Fox 2011; Zugai et al. 2012). On the contrary, in situations when nurses failed to demonstrate such commitment and genuineness, it resulted in a sense of ‘us versus them’ (Pemperton and Fox 2011). An open and genuine interest for the patients’ difficulties which was based on patient-focused conversations was highlighted in several studies as important for the relationship (Gulliksen et al. 2012; Offord et al. 2006; Sly et al. 2014). If the patients were able to share their thoughts and feelings with the nurses, the conversation was thought to help the patient forward in the health process (Bakker et al. 2011).

#### Trust and Safety

Nurses cited trust as important in the care of people with AN, but also for guiding a change in the patients’ behaviours (van Ommen et al. 2009; Zugai et al. 2012). Trust was described as a component of the relationship and could be developed over time (Gulliksen et al. 2012; Micevski and McCann 2005). Patients’ feelings of trust in the relationship grew as the nurses demonstrated knowledge of the illness (Ross and Green 2011). On the contrary, Ramjan (2004) described that when nurses lacked knowledge about the illness, this mindset facilitated an overly narrowed focus on the patient’s behaviours, resulting in a power struggle in the relationship. Patients were considered to be manipulative, and nurses felt that patients saw them as enemies with authoritative approaches, and the interventions were considered to be punitive.

### The Person at the Centre

This theme rests on the ability for nurses to separate the diagnosis of a patient from them as individuals. Studies suggest that nurses must not prioritise the physical aspects of the illness over the psychological. The two sub-themes are woven to one theme: seeing the person behind the diagnosis and finding a balance between physical and psychological issues.

#### Seeing the Person Behind the Diagnosis

Several studies emphasised that both nurses and patients found it imperative that nurses clearly distinguish the two-fold view of the patient’s identity, as including both the patient’s diagnosis and the patient as an individual (Colton and Pistrang 2004; Gulliksen et al. 2012; Jenkins and Ogden 2011; Micevski and McCann 2005; van Ommen et al. 2009; Ross and Green 2011; Sly et al. 2014; Tierney 2008; Wright and Hacking 2012). However, this is not always easy, as demonstrated in Ramjans’ (2004) study, where nurses expressed negative experiences as they viewed patients with anorexia as manipulative and distrustful. Nurses who behaved in an authoritarian manner and were seen as prejudiced because they put the diagnosis before the individual provoked a feeling of loss of identity, and made the patients feel as ‘just another anorexia case’ (Gulliksen et al. 2012; Tierney 2008). This was thought to reinforce the illness and the identity of being anorexic. When nurses were able to express respect for the person behind the illness, it helped the patients to take a more active role in the relationship, and these patients were able to progress toward health (Micevski and McCann 2005). Sly et al. (2014) stressed that a strategy among nurses was to let the patients tell their own stories.

#### The Balance between the Physical and the Psychological

Several studies stressed the nurses’ inability to pay attention to patients’ needs for psychological support to the same extent as they did to the physical issues. Such a single-minded focus on weight reinforced the feelings among patients of being their diagnosis (Colton and Pistrang 2004; Jenkins and Ogden 2011; Offord et al. 2006; Pemperton and Fox 2011; Tierney 2008). The unbalanced focus hampered the relationship as patients perceived that the nurses did not want to be supportive of all their needs but, rather, saw only the goal of the patients reaching a certain weight (Pemperton and Fox 2011). The feeling of loss of control due to weight gain and lack of psychological support strengthened the anorexic behaviours, thereby working against the patient’s health process (Offord et al. 2006; Tierney 2008).

### Nurses’ Attitudes

One prominent theme addressed the personae of the nurses and the way nurses approached the patients. These aspects were seen as keys to promoting a relationship and helping patients in their processes of recovery. The nurse was described as a role model regarding establishing norms, responsibilities and structure in the patient’s life. Nurses’ attitudes is the overarching theme for the following three sub-themes: motivation and hope, maintaining structure, responsibility and normality, and presence and availability.

#### Motivation and Hope

Nurses play an important role in informing and educating the patients about the treatment and therapeutic goals in order to motivate the patients. Bakker et al. (2011) emphasises the need for nurses to persuade patients that interventions and restrictions are actions against the illness and not against the patient as a person. In several studies, motivation was seen as pivotal when hope and optimism wavered, as was the way in which nurses presented challenges, rules and restrictions in daily life (Colton and Pistrang 2004; Gulliksen et al. 2012; Offord et al. 2006; van Ommen et al. 2009; Sly et al. 2014; Wright and Hacking 2012; Zugai et al. 2012). However, restrictions as treatment interventions had to feel relevant and individually adjusted (Zugai et al. 2012), otherwise they were understood to be punitive and not strengthened (Colton and Pistrang 2004; van Ommen et al. 2009; Zugai et al. 2012). The nurse was viewed as a leader and a role model who made it possible to move forward in the process of recovery. On the other hand, the feeling of not being backed by the nurse evoked a feeling of resignation (Sly et al. 2014). Furthermore, Wright and Hacking (2012) emphasised the importance of the nurse in the therapeutic relationship, as the nurse was described as a “saviour” that helped patients move away from the disease.

#### Maintaining Structure, Responsibility and Normality

In the early stages of recovery, when patients’ compulsive behaviours were most intense, the nurses advocated for a structured approach (Bakker et al. 2011; van Ommen et al. 2009; Ross and Green 2011; Zugai et al. 2012). Patients considered it to be helpful when nurses took over responsibility for food intake when anorexic thoughts and behaviours became too strong, and maintained boundaries and structure in order to establish normality (Offord et al. 2006; van Ommen et al. 2009; Ross and Green 2011; Zugai et al. 2012). However, as nurses took control from the patients, it was considered essential that they clearly demonstrated empathy and understanding for the patients’ feelings (Bakker et al. 2011; van Ommen et al. 2009). When nurses were understanding but at the same time confident enough to challenge the patient, this was experienced positively and it strengthened the feeling that the nurses were there for them (Gulliksen et al. 2012).

#### Presence and Availability

Several studies emphasised that a nurse’s presence and availability were essential for the relationship (Offord et al. 2006; van Ommen et al. 2009; Wright and Hacking 2012). Patients in the study conducted by Colton and Pistrang (2004) believed that continual emotional support, such as short daily conversations, was better than scheduled, weekly therapy. In the study by van Ommen et al. (2009), patients described the importance of knowing that nurses were “emotionally available” and that they could distinguish patients’ feelings and were willing to devote time to them. Furthermore, along the later phase in the recovery process, it was necessary that the nurses were able to find a balance between closeness and distance in order to help the patient move forward and make more independent choices concerning food intake and meals (Bakker et al. 2011; van Ommen et al. 2009). As the nurses became less present in the later phases, patients felt motivated and safe and this strengthened the patients’ self-esteem and feelings of independence (Offord et al. 2006). Nevertheless, as nurses’ presence decreased this was considered to be associated with an increased risk of remission as the patients were released (Bakker et al. 2011; van Ommen et al. 2009).

### Knowledge

The last theme includes the nurses’ knowledge and understanding of the illness but also concerns the patients’ insights into the illness as crucial aspects of the relationships. The theme addresses aspects such as knowledge and understanding and the ability to manage and identify feelings. This included the nurse’s ability to provide the patients with strategies to manage their own feeling, to provide support to patients and to help them to identify, understand and manage their own feelings. The theme was broken down into two sub-themes: understanding, experience and knowledge; and emotional management and identification.

#### Understanding, Experience and Knowledge

Lack of knowledge about the illness often leads to inconsistency and ambiguity in the relationship (Micevski and McCann 2005)-in contrast to the consistency and structure that otherwise was considered important for the relationship (van Ommen et al. 2009). Collegial interactions and sharing relational issues with other nurses based upon their experience and knowledge was found to be perceived as helpful in order to develop relationships with patients. Lack of support from colleagues was thought to hamper relationships with the patients (Micevski and McCann 2005). The aspect of nurses experiences was also viewed from the patient’s perspective, as demonstrated in the study by Tierney (2008). Lack of knowledge could result in a perception among nurses that the patients themselves were responsible for the illness and hence should be able to ‘fix themselves’ (Ramjan 2004). Such attitudes toward eating disorders entailed that the nurses performed routine behaviour and control work. In turn, the patients were perceived as rebellious, which reinforced the power struggle and hampered the relationship. In contrast, Bakker et al. (2011) stressed the importance of being able to demonstrate an understanding of the illness but at the same time emphasised that nurses must have the knowledge and ability to openly discuss issues. As a consequence of nurses’ lack of knowledge about anorexia, the acute divest of patients’ control of meals resulted in power structures that extended into other areas of the patients’ lives. Due to the lack of understanding and empathy from nurses, the interventions in treatment were perceived more punitively, resulting in a worse relationship (Offord et al. 2006).

#### Emotional Management and Identification

The nurses’ abilities to provide patients with strategies to manage their own emotions was yet another key aspect in a supportive relationship that allowed for the patients to move forward in the process (Pemperton and Fox 2011; Ross and Green 2011). The relationship with nurses was associated with a sense of being protected and being provided with support, as nurses had not only the ability to identify the patient’s feelings but also were able to validate and name them for the patient. (Jenkins and Ogden 2011; Pemperton and Fox 2011; Ross and Green 2011; Zugai et al. 2012).

The nurses’ reactions to the patients’ displays of feelings, particularly aggression, were deciding factors in whether patients could rely on them for help in the future. On the other hand, a difficulty with identifying and showing feelings, together with mistrust, contributed to more negative emotions -for example, the feeling of being abandoned-as well as a larger degree of suppressed feelings. This was eventually considered to act as a “trigger” and contribute to a further increase in anorexic behaviours (Pemperton and Fox 2011).

In Ross and Green’s (2011) study, the nurses abilities to handle the patients’ emotions were seen as having the potential to affect the patients’ senses of security and belonging, which were previously described as central for a therapeutic relationship. The nurses’ abilities to understand and respond to the patients’ feelings with empathy and interpret the patients’ feelings were described as having supportive and nurturing effects. This contributed to the patients’ awareness of the disease and their understandings of past experiences, which affected the process toward better health (Ross and Green 2011; Zugai et al. 2012). The patients felt that the personal contact with the responsible nurses was the most helpful, as this gave ongoing validation and emotional support to the patient (Offord et al. 2006).

## Discussion

In this review, we conducted a descriptive literature synthesis in which we reviewed 14 qualitative study articles in order to identify and describe factors that promote and impede the relationships between nurses and the children, adolescents and young adults who are diagnosed with AN. There is not yet enough evidence to reach a consensus about best practices and the gold standard of treatment (Marzola et al. 2012), and our review does not succeed to provide an answer to the golden standard either. Earlier studies have acknowledged that emotional alliances and trusting relationships is essential in psychiatric care (Borg and Kristiansen 2004; Denhov and Topor 2012), but in addition demonstrated the difficulties in creating an alliance with AN diagnosed patients (Pereira et al. 2006). This review has explicitly accounted on describing nurses’ establishment of health promoting relationships and contribute with important insights to the body of knowledge in this area of the literature. Our main findings demonstrate that the nurses first and foremost have to establish relationships that are characterised by feelings of solidarity, participation, equality, openness, sincerity and honesty and that they are able to convey a sense of trust and safety. These qualitative interpersonal values were understood as prerequisites for the coming therapy. The importance of establishing an alliance between patients and their caregivers is one key aspect in the recovery process (Bell 2003; De la Rie et al. 2006; Masson and Sheeshka 2009). Consistent with our key findings, previous research have reported that a relationship is necessary if the patient is to view the care as meaningful. We acknowledge that establishing a good relationship with patients who are diagnosed with AN is complex and nonlinear. From our findings we can conclude that nurses must value their efforts to commit to establishing and maintaining relationships with patients. In this sense, nurses’ expertise must go beyond linear thinking, according to pathophysiology and bio-physiological explanation models (cf. Coppa 1993).

The tenets of establishing a health-promoting relationship between nurses and patients has been stressed in nursing theories (Dahlberg and Segesten 2010; Peplau 1991). Even though relational aspects are important, we advocate for a more detailed theoretical understanding of patients with AN and their specific needs. Our findings have provided more far-reaching insights into strategies that can be adopted by nurses. This review also adds an understanding of how nurses’ attitudes may foster the relationship and have an impact on their abilities to motivate the patients with hope, a sense of their own responsibility and better knowledge in emotional management. We emphasise that those concepts in the findings are similar to concepts in recovery-models in mental health care. Anthony (1993) portrayed recovery to be a deeply personal process of changing attitudes, values and feelings in order to develop new meaning and purpose in one’s life beyond the effects of mental illness. We stress the necessity of nurses that are able to balance their roles as leaders, role models, and those that maintain boundaries and structure, but they also must acknowledge the patient’s own capabilities and step aside to leave the patient with responsibility. When doing so, we see the potential to develop a caring attitude that is in line with Anthony’s conceptualization.

In order to further theorise on the nurse as a companion in the recovery process of the patient, we stress that aspects that promote and impede the relationship have been given less priority in research. Our results affirm that nurses, from their approaches, highly influence whether the relationship will promote or impede the patient’s health progress. A key aspect in the relationship was the nurses’ views of the patients and what the nurses wanted to accomplish with treatment. Nurses who view the patients as cases demonstrated a standardised response and interest, where the diagnosis was in the foreground and the goal was mainly to ensure weight gain. They made generalisations about the patient’s feelings and actions based on stereotypical prejudices toward the patient’s symptoms, which could reinforce the identity of being anorexic and impede the process toward better health (Gulliksen et al. 2012; Offord et al. 2006). We draw parallels between nurses’ behaviours and Plummer’s (1979) description of secondary deviance, as nurses not only adopt a standardized approach and view patients as cases, but they also become responsible for a more far-reaching process in which the nurses also have the potential to negatively influence and impede recovery and gradually build a deviant self. This process of stigmatizing patients is the opposite of Anthony’s (1993) idea of personal recovery process. Hence, we stress the importance of understanding the complex mechanisms that reach beyond the patient’s manifestations of symptoms in order to overcome negative spirals in the relationships. Furthermore, in several studies patients expressed that the one-dimensional view from professional theories were thought to give rise to expectations and prejudices based on general experience and assumptions as well as nurses’ own insecurities, which wasn’t experienced as supporting the progress toward better health (Jenkins and Ogden 2011; Offord et al. 2006; Pemperton and Fox 2011; Wright and Hacking 2012).

It was helpful when nurses were able to put the patients’ own motivations to be well at the centre of the care in order to build an alliance, a finding which is in accordance with previous studies (Kaplan and Garfinkel 1999; Westwood and Kendal 2011). The results emphasise that it is only when the nurse shows a genuine interest in the patient and has a deeper understanding of the patient’s life as well as when the patient perceives collaboration with the nurses that trust is born–the basis for a relationship. One study conducted by Topor et al. (2006) demonstrated how professional roles may contribute to personal recovery. It was reported that when professionals depart from their formal roles, they risk to be criticised by colleagues, but, more importantly, such departures are view as meaningful and contribute to the patient’s personal recovery (Topor et al. 2006). In this sense, we accentuate the value of the nurses’ will and courage to care in favour of the interests of the patients. We posit that it is necessary for nurses to reflect on and translocate the ontological stance in nursing from caring for to caring with (cf. Barker and Buchanan-Barker 2005).

Lastly, we will discuss the methodological considerations. A broad range of search terms were used in three databases, all of which were highly relevant for nursing research and for the aim of this review. In addition to the search terms, free searches and manual searches were conducted. At the end of the search process, the authors repeatedly found the same articles, which we interpreted to indicate that no more relevant articles could be found. Alternative search terms might have yielded more studies. All included articles were evaluated based on quality, and several articles were excluded due to low quality. One author who was well-familiarised with the process of evaluating research articles evaluated the quality, although no assessment of inter-rater reliability was performed. The second author was mainly responsible for the analysis process. However, throughout the analysis process, both authors engaged in frequent discussions about the coding procedure, examining individual codes and their accuracy within specific categories as well as the labelling of categories and themes. Even though this exclusion of low quality articles gave more trustworthiness to our review, we might have missed aspects that were not reported in our findings. Since the purpose of our review was to describe the phenomena from both nurses and patients with AN, we recognise an imbalance between the two categories, which suggests a rationale for conducting future studies only from one perspective. All included articles were also conducted in the Western World, in countries with similar health care systems. This notion might have skewed the results. Surprisingly, we observed that the results in the 14 articles were strikingly similar even though the demographics of the included participants differed in age, length of illness and treatment forms (both in and out-patient facilities). The results from qualitative inquiries do not have external validity, and Creswell (2007) argues for transferability as a measure of trustworthiness. As such, we do not claim that the findings are applicable in other healthcare settings than the studies.

## Electronic supplementary material

Supplementary Table1

Supplementary Table2

Supplementary Table3
