# Supplementary material for: Nurses’ Establishment of Health Promoting Relationships: A Descriptive Synthesis of Anorexia Nervosa Research
Source: J Child Fam Stud. 2016 Sep 13;26(1):1–13. doi: 10.1007/s10826-016-0534-2 (PMC5219017; doi:10.1007/s10826-016-0534-2)
Supplement: Supplementary file 1 — Supplementary Table1 [file 10826_2016_534_MOESM1_ESM.docx]

Supplementary Materials

**Table 1 (Supplemental)** Search matrix

| **Database CINAHL** |  |  |  |  |  |  |  |  |
| --- | --- | --- | --- | --- | --- | --- | --- | --- |
| **Manual search** | **Search word** | **Number of hits** | **Titles read** | **Abstracts read** | **Overlapping abstracts** | **Articles reviewed** | **Articles chosen** | **Article** |
| 1 | Anorexia | 4 859 | 0 |  |  |  |  |  |
| 2 | Qualitative | 73 041 | 0 |  |  |  |  |  |
| 3 | Nurse* | 522 651 | 0 |  |  |  |  |  |
| 4 | Relationship* | 310 998 | 0 |  |  |  |  |  |
| 5 | Therapeutic alliance | 645 | 0 |  |  |  |  |  |
| 6 | Effective nursing* | 17 818 | 0 |  |  |  |  |  |
|  |  |  |  |  |  |  |  |  |
|  | 1 + 2 | 144 | 144 | 17 | 0 | 15 | 9 | B,C,D,E,F,G, J,L, M. |
|  | 1 + 3 | 354 | 354 | 0 |  |  |  |  |
|  | 1 + 4 | 750 | 750 | 0 |  |  |  |  |
|  | 1 + 5 | 23 | 23 | 5 | 1 | 0 |  |  |
|  | 1 + 6 | 37 | 37 | 4 | 4 | 0 |  |  |
|  | 1 + 3 + 4 | 75 | 75 | 3 | 2 | 0 |  |  |
|  | 1 + 4 + 5 | 14 | 14 | 4 | 3 | 0 |  |  |
|  | 1 +2 + 3 + 4 | 11 | 11 | 1 | 0 | 1 | 1 | I |

Date: 10-21-2014. Overlapping abstracts = abstracts that were already read in earlier searches and therefore not read again.

| **Database  CINAHL** |  |  |  |  |  |  |  |  |
| --- | --- | --- | --- | --- | --- | --- | --- | --- |
| **Search by subject headings** | **Search words** | **Number of hits** | **Titles read** | **Abstracts read** | **Overlapping abstracts** | **Articles reviewed** | **Articles chosen** | **Article** |
| 1 | Anorexia Nervosa | 3 171 | 0 |  |  |  |  |  |
| 2 | Qualitative studies | 62 286 | 0 |  |  |  |  |  |
| 3 | Nursing care | 168 432 | 0 |  |  |  |  |  |
| 4 | Interpersonal relationships | 23 471 | 0 |  |  |  |  |  |
| 5 | Professional-patient relationships | 25 729 | 0 |  |  |  |  |  |
|  |  |  |  |  |  |  |  |  |
|  | 1+ 2 | 109 | 109 | 4 | 12 | 4 | 1 | K |
|  | 1+ 3 | 52 | 52 | 0 | 1 | 0 | 0 |  |
|  | 1+ 4 | 59 | 59 | 1 | 3 | 1 | 1 | A |
|  | 1+ 5 | 37 | 37 | 1 | 5 | 1 | 0 |  |

Date: 10-22-2014. Overlapping abstracts = abstracts that were already read in earlier searches and therefore not read again.

| **Database PsycINFO** |  |  |  |  |  |  |  |  |
| --- | --- | --- | --- | --- | --- | --- | --- | --- |
| **Manual search** | **Search word** | **Number of hits** | **Titles read** | **Abstracts read** | **Overlapping abstracts** | **Articles reviewed** | **Articles chosen** | **Article** |
| 1 | Anorexia Nervosa | 8825 | 0 |  |  |  |  |  |
| 2 | Nursing | 79 708 | 0 |  |  |  |  |  |
| 3 | Therapeutic relationships* | 24 297 | 0 |  |  |  |  |  |
| 4 | Professional relationships* | 42 205 | 0 |  |  |  |  |  |
| 5 | Alliance | 9 290 | 0 |  |  |  |  |  |
| 6 | Effective nursing* | 7 836 | 0 |  |  |  |  |  |
|  |  |  |  |  |  |  |  |  |
|  | 1+ 2 | 118 | 118 | 2 | 4 | 1 | 1 | H |
|  | 1+3 | 177 | 177 | 2 | 7 | 2 | 0 |  |
|  | 1+4 | 45 | 45 | 1 | 4 | 1 | 0 |  |
|  | 1+5 | 74 | 74 | 4 | 7 | 0 | 0 |  |
|  | 1+6 | 9 | 9 | 0 | 3 | 0 | 0 |  |
|  |  |  |  |  |  |  |  |  |
|  |  |  |  |  |  |  |  |  |

Date: 10-23-2014. Overlapping abstracts = abstracts that were already read in earlier searches and therefore not read again.

| **Database  PsycINFO** |  |  |  |  |  |  |  |  |
| --- | --- | --- | --- | --- | --- | --- | --- | --- |
| **Search with Thesaurus** | **Search word** | **Number of hits** | **Titles read** | **Abstracts read** | **Overlapping abstracts** | **Articles reviewed** | **Articles chosen** | **Article** |
| 1 | Anorexia Nervosa | 8 825 | 0 |  |  |  |  |  |
| 2 | Qualitative research | 37 138 | 0 |  |  |  |  |  |
| 3 | Therapeutic process* | 38 064 | 0 |  |  |  |  |  |
| 4 | Interpersonal relationship | 34 809 | 0 |  |  |  |  |  |
|  |  |  |  |  |  |  |  |  |
|  | 1+2 | 83 | 83 | 0 | 6 | 0 | 0 |  |
|  | 1+3 | 187 | 187 | 2 | 5 | 2 | 0 |  |
|  | 1+4 | 80 | 80 | 0 | 2 | 0 | 0 |  |

Date: 10-23-2014. Overlapping abstracts = abstracts that were already read in earlier searches and therefore not read again

| **Database PubMed** |  |  |  |  |  |  |  |  |
| --- | --- | --- | --- | --- | --- | --- | --- | --- |
| **Search with MeSH terms** | **Search words** | **Number of hits** | **Titles read** | **Abstracts read** | **Overlapping abstracts** | **Articles reviewed** | **Articles chosen** | **Article** |
| 1 | Anorexia Nervosa | 13 059 | 0 |  | 0 |  |  |  |
| 2 | Nurse -patient relationship* | 31 586 | 0 |  | 0 |  |  |  |
| 3 | Professional-patient relationship* | 35 434 | 0 |  | 0 |  |  |  |
| 4 | Qualitative research | 92 038 | 0 |  | 0 |  |  |  |
|  |  |  |  |  |  |  |  |  |
|  | 1+ 2 | 56 | 56 | 1 | 10 | 0 | 0 |  |
|  | 1+3 | 193 | 193 | 1 | 15 | 0 | 0 |  |
|  | 1+4 | 105 | 105 | 2 | 12 | 1 | 0 |  |
|  |  |  |  |  |  |  |  |  |

Date: 25-10-2014. Overlapping abstracts = abstracts that were already read in earlier searches and therefore not read again.

**Manual search:**Articles from the found articles’ reference lists were also searched manually. Seven abstracts were read.
One article was reviewed and thereafter chosen to be included in the study (N).
