# Supplementary material for: Nurses’ Establishment of Health Promoting Relationships: A Descriptive Synthesis of Anorexia Nervosa Research
Source: J Child Fam Stud. 2016 Sep 13;26(1):1–13. doi: 10.1007/s10826-016-0534-2 (PMC5219017; doi:10.1007/s10826-016-0534-2)
Supplement: Supplementary file 2 — Supplementary Table2 [file 10826_2016_534_MOESM2_ESM.docx]

Supplementary Materials

Table 2 (Supplemental Material) Screening Template for Determining Quality, Qualitative Studies

|  | **Question** | Yes | No |
| --- | --- | --- | --- |
| 1 | Does the study’s title correspond with its content? |  |  |
| 2 | Does the abstract of the study represent the content? |  |  |
| 3 | Does the introduction provide an adequate description of the selected investigation? |  |  |
| 4 | Does the introduction logically lead to the study’s objective? |  |  |
| 5 | Is the study’s purpose clearly formulated? |  |  |
| 6 | Is the qualitative method described clearly? |  |  |
| 7 | Is the design relevant based on the goal? |  |  |
| 8 | Are the criteria for inclusion described? |  |  |
| 9 | Are the criteria for inclusion relevant? |  |  |
| 10 | Are the criteria for exclusion described? |  |  |
| 11 | Are the criteria for exclusion relevant? |  |  |
| 12 | Are the selection criteria described? |  |  |
| 13 | Are the selection criteria relevant to the study’s purpose? |  |  |
| 14 | Is the study group described with regards to background variables? |  |  |
| 15 | Does the article indicate where the study was conducted? |  |  |
| 16 | Does the article indicate when the study was conducted? |  |  |
| 17 | Is the selected data collection method stated? |  |  |
| 18 | Is the data systematically collected? |  |  |
| 19 | How has the data been analysed? |  |  |
| 20 | Are the results credibly described? |  |  |
| 21 | Is the study’s purpose answered? |  |  |
| 22 | Are the conclusions that can be drawn from the study’s results described? |  |  |
| 23 | Is the study’s credibility discussed? |  |  |
| 24 | Are the study’s ethical aspects discussed? |  |  |
| 25 | Is the study’s clinical value discussed? |  |  |
| Totals: |  |  |  |

Maximum points: 25

Quality levels
High: 21-25 points
Medium: 18-20 points
Low: 0-17 points


The template is a modified version of Willman, A., Stoltz, P., & Bathsevani, C (2006) and Forsberg, C., & Wengström, Y (2013)
