# Supplementary material for: Nurses’ Establishment of Health Promoting Relationships: A Descriptive Synthesis of Anorexia Nervosa Research
Source: J Child Fam Stud. 2016 Sep 13;26(1):1–13. doi: 10.1007/s10826-016-0534-2 (PMC5219017; doi:10.1007/s10826-016-0534-2)
Supplement: Supplementary file 3 — Supplementary Table3 [file 10826_2016_534_MOESM3_ESM.docx]

Supplementary Materials

**Table 3 (Supplemental)** Excerpts from the Matrix with Coding and Themes

| Key finding, # 1  Key finding, # 4 |  | Sub-theme 1: Motivation and hope |
| --- | --- | --- |
| Key finding, # 18 Key finding… | Main theme: Nurses’ attitudes | Sub-theme 2: maintaining structure, responsibility and normality |
| Key finding… |  | Subtheme 3: Presence and availability |
| Key finding, # 14 Key finding, # 26 |  | Sub-theme 4: Feelings of solidarity, participation and equality |
| Key finding, # 107  Key finding… | Main theme: The Essentials in a Relationship | Sub-theme 5: Openness, integrity and honesty |
| Key finding… |  | Sub-theme 6: Trust and safety |

|  | **Article, author (participants)** | **Key findings from the article** | **Abbreviated key findings on post-it notes** | **Main theme** | **Sub-themes** |
| --- | --- | --- | --- | --- | --- |
| 1 | Zugai et al. (2012)  (patients) | Relinquishing control involved nurses implementing strict  rules in order to ensure adherence to prescribed eating and exercise patterns. | Nurses’ taking over of control of food and movement. *A way to relate and work..*( 1) | Nurses’ attitudes | Structure, responsibility and normality |
| 4 | Zugai et al. (2012)  (patients) | rules of the eating disorder programme had to be relevant, fair and unrestrictive. Participants reflected negatively about nurses that were seen as too strict or too rigorous. The participants expressed that nurses should apply rules in ways that take into account the intent of the rules rather than an inflexible approach. Rules that did not have clear weight gain measures or health outcomes were perceived as punitive. | Stressing the importance that nurses could *motivate for restrictions*, which otherwise were experienced as punishments. Nurses’ way of working. (4) | Nurses’ attitudes | Motivation and hope |
| 14 | Wright & Hacking (2012)  (nurses) | Both staff members and patients expressed the need to be genuine and transparent in order to establish an authentic and therapeutic relationship. | Confidence, that the patient dares to speak up, an *openness between nurse and patient*. *Good for relationship.* (14) | The Essentials in a Relationship | Openness, integrity and honesty |
| 18 | Wright & Hacking (2012)  (patients) | Nurse in terms of a life raft…// the process of sharing the experience of the disorder …//(nursing as) a safe place, somewhere to go together with the patient. | *The nurses’ ways of being,* to always be there for the patient. Strengthens the relationship. (18) | Nurses’ attitudes | Presence and availability |
| 26 | Offord et al. (2006)   (patients) | The perception that services were uncollaborative, rigid and controlling led some participants to fight back, perhaps hindering their recovery…/successful in-patient treatment was reported by those who described a strong sense of being involved in their own care. | Low levels of cooperation – bad for relationship. *Cooperation between nurses and patients involvement in their own care good.* (26) | The Essentials in a Relationship | Feelings of solidarity, participation and equality |
| 47 | Pemperton & Fox (2011)   (patients) | Management of difficult or negative emotions was dependent upon the staff’s perceived understandings of both the patient’s emotions as well as their own. /staff members’ perceived abilities to reflect upon and use their own past personal emotions to understand the participant’s emotional experiences. | Understanding of both the patient’s feelings as well as the nurses’ own. *Ability to manage feelings.*  (47) | Knowledge | Handing feeling and identification |
| 48 | Pemperton & Fox (2011)  (patients) | Individual differences among staff may factor into why some staff may depersonalize patients more than others. Age, personality, experience and intelligence, for example, could all impact the need to feel a strong alliance with the in-group, which could strengthen the perceived ‘difference’ of the out-group. | “Us vs. them”. Nurses’ characteristics play into the ability to see the difference between the person and the diagnosis*.* (48) | The person at the centre | Seeing the person behind the diagnosis |
| 107 | Ramjan (2004)  (nurses) | Manipulation, distrust and the struggle for control were the major obstacles in developing therapeutic relationships in these wards. | Distrust and power struggles between nurses and patients, *obstacles for relationships.* (107) | The Essentials in a Relationship | Trust and safety |
| 108 | Ramjan (2004)  (nurses) | In the absence of real understandings about anorexia or the recovery process, participants saw their work in terms of the behaviour modification programme that they were required to enforce. The extremely controlling nature of the programme led to rebellion from patients, who were then perceived as manipulative. | *Incompetence and lack of understanding* of the anorexia. Focus on behaviour. (108) | Knowledge | Understanding, experience and knowledge |
